# Clinicopathologic Relevance of Claudin 18.2 Expression in Gastric Cancer: A Meta-Analysis

**DOI:** 10.3389/fonc.2021.643872

**Published:** 2021-03-04

**Authors:** Bogdan Silviu Ungureanu, Cristian-Virgil Lungulescu, Daniel Pirici, Adina Turcu-Stiolica, Dan Ionut Gheonea, Victor Mihai Sacerdotianu, Ilona Mihaela Liliac, Emil Moraru, Felix Bende, Adrian Saftoiu

**Affiliations:** ^1^Gastroenterology Department, University of Medicine and Pharmacy of Craiova, Craiova, Romania; ^2^Oncology Department, University of Medicine and Pharmacy of Craiova, Craiova, Romania; ^3^Histology Department, University of Medicine and Pharmacy of Craiova, Craiova, Romania; ^4^Pharmacoeconomics Department, University of Medicine and Pharmacy of Craiova, Craiova, Romania; ^5^Surgical Department, University of Medicine and Pharmacy of Craiova, Craiova, Romania; ^6^Gastroenterology Department, University of Medicine and Pharmacy “Victor Babes”, Timisoara, Romania

**Keywords:** claudin 18.2, gastric cancer, TNM stages, HER2, Lauren classification

## Abstract

An increasing number of tumor markers have been discovered to have potential efficacy as diagnostic and prognostic tools in gastric cancer. We aimed to assess putative correlations between claudin 18.2 expression and pathological or prognosis features in patients with gastric cancer. MEDLINE, Web of Science, EBSCO, and ClinicalTrials.gov were used to search for relevant studies from their inception to 30 October 2020. Finally, a total of six articles were included in this meta-analysis. Review Manager 5 software was applied to examine the heterogeneity among the studies and to calculate the odds ratio with 95% CI by selecting corresponding models, in evaluating the strength of the relationship. Publication bias test was also conducted. No bias and no significant correlations were found between CLDN 18.2 and TNM stages, Lauren classification, HER2, grading, or overall survival. This meta-analysis expounded that the relationship with CLDN 18.2 and pathological features depends on the percentage of staining of tumor cells for which CLDN 18.2 is considered positive. Our pooled outcomes suggest that targeted therapy for CLDN 18.2 could be effective if certain criteria were established.

## Introduction

Gastric cancer (GC) is one of the most commonly diagnosed malignancies worldwide and the second cause of cancer-related death. Despite the variability of GC incidence and mortality, an estimated 1,033,701 new stomach cancers and 782,685 deaths occurred in 2018^1^. Frequently, patients are diagnosed at an advanced stage, especially in countries where GC screening is not routinely performed, aggravating its poor prognosis.

Targeted agents approved for GC like trastuzumab (anti-HER2) or ramucirumab (anti-VEGF receptor) have shortcomings such as modest survival benefits and second resistance development. New suitable biomarkers that can serve as targets have to be found for highly effective targeted therapies for GC (1).

Claudins are a family of at minimum 27 proteins with roles in maintaining the intercellular tight junction adhesion, which create a paracellular barrier. The impossibility of these molecules to accomplish their function is linked with tumor development and progression (2, 3). Different claudins expression may have prognostic value in colon cancer [claudin (CLDN)-1] (4), pancreatic cancer (CLDN-18), and hepatocellular carcinoma and thyroid cancer (CLDN-10) (5, 6). CLDN 18 has two isoforms (CLDN 18.1 and CLDN 18.2), which are present in differentiated epithelial cells of gastric mucosa. CLDN 18 splice variant 2 is the dominant isoform that occurs in normal gastric tissue, gastric adenocarcinomas, and their metastases. Furthermore, CLDN 18.2 is aberrantly expressed in pancreatic, esophageal, ovarian, and lung adenocarcinomas (7). CLDN 18.2 is an attractive surface biomarker as it is located on the outer cell membrane, therefore easy accessible for targeted therapies (8).

IMAB362 (known as zolbetuximab or claudiximab), a novel chimeric immunoglobulin G1 antibody, is the first type of ideal monoclonal antibodies (IMAB) used for the treatment of GC. After IMAB362 binds to CLDN 18.2, immune effectors activate antibody-dependent cellular cytotoxicity and complement-dependent cytotoxicity. This change induces apoptosis and promotes the inhibition of cell proliferation, with beneficial effects for patients (9).

Our objective was to assess all available studies that involve CLDN 18.2 expression in GC and its relation to clinicopathological or prognosis features in patients with GC, in order to offer more insights on its potential as a target in future clinical trials.

## Materials and Methods

### Literature Search

We used the PICOS (populations, interventions, comparators, outcomes, and study designs) model and PRISMA guidelines to design our search strategy (10).

To identify studies, we searched the following databases: MEDLINE, Web of Science, EBSCO, and ClinicalTrials.gov (inception to 30 October 2020) to see if they evaluated the expression of CLDN 18.2 in order to find correlations with clinicopathological patient characteristics with GC. We studied reference lists as well as published systematic review articles. The search terms included (“claudin 18.2” AND “gastric cancer”) OR (“claudin18.2” AND “gastric cancer”).

### Inclusion and Exclusion Criteria

Studies evaluating the expression of CLDN 18.2 in adults with GC were included in our meta-analysis. The inclusion criteria for selection were: (1) clear definition of scoring for CLDN 18.2 staining; (2) assessment of clinicopathological patient characteristics; (3) histologically confirmed adenocarcinoma of the stomach. Exclusion criteria were: (1) tumor types other than adenocarcinoma; (2) patients who had undergone a perioperative or neoadjuvant chemo- or radiotherapy; (3) studies as case reports, systematic reviews, abstracts.

### Data Extraction and Quality Assessment

Two review authors (BSU and VMS) independently extracted all data using a standardized data extraction table. Any disagreements regarding eligible articles were resolved after consulting a third review author (AT-S). The risk of bias was assessed through a funnel plot.

### Statistical Analysis

We conducted a standard meta-analysis using the Review Manager 5 software (RevMan 5. Version 5.4.1, the Cochrane Collaboration, 2020). We used both the random-effects model and the fixed-effects model based on the assessment of heterogeneity, when the inverse-variance approach was implemented. We used the I^2^ statistic, which gave us the proportion of the observed variance that reflects real differences in effect size, for quantifying heterogeneity of the results in individual studies, which combined the Chi^2^ statistic and the number of studies contributing to each summary estimate in the forest plot (11).

We used odds ratio (OR) as the effect measure for dichotomous outcomes, that is the number of participants achieving TNM clinical stage, HER2, Lauren classification, and grading. Analysis and comparisons for all outcomes were performed where data were available. We considered *P*-values <0.05 and 95% confidence intervals (CI) that did not include 1 to be statistically significant.

Time-to-event data for overall-survival (OS) were analyzed using hazard ratio (HR), which was estimated using the calculation methods described by Tierney et al. (12). If these parameters were not available in the studies, we used WebPlot Digitizer version 4.3 (Austin, Texas, USA) to extract the specific survival rates from the Kaplan-Meier curves.

To assure our results were robust, the presence of any publication bias was analyzed with a funnel plot, based on the visual inspection of the symmetry.

## Results

**Figure 1:**
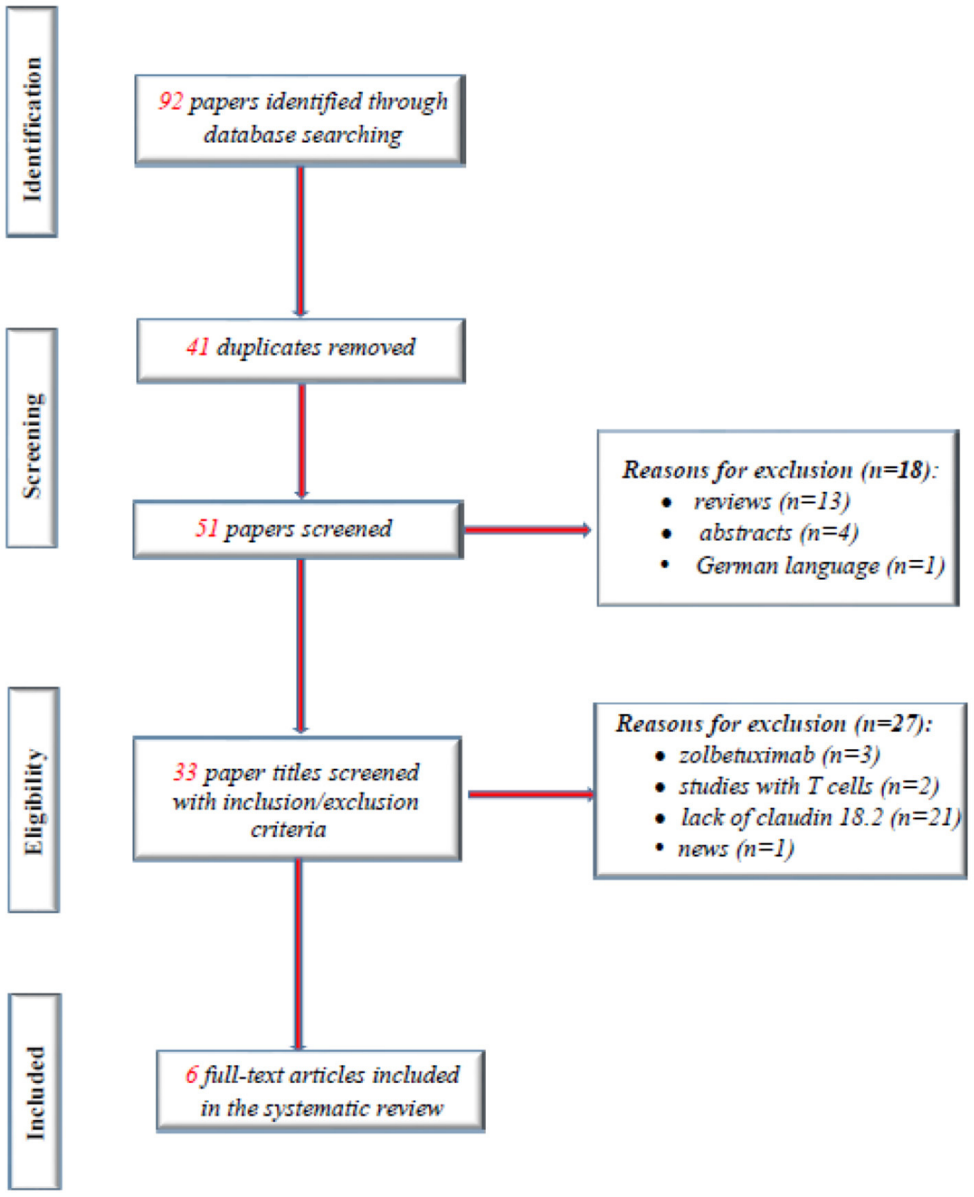
Study flow diagram.

Figure 1 shows the overall study selection process. We identified a total of six eligible studies, including 2,440 patients. A total of 86 studies were excluded, and the main reasons for exclusion included lack of information about the correlation of CLDN 18.2 expression and clinicopathological patient features and duplicate studies or abstracts.

### Baseline Characteristics of All Included Studies

The characteristics of the included studies are provided in Table 1. The study sample size ranged from 263 to 485 participants. The six studies revealed a prevalence of 34.2% from the total of 2,055 patients.

**Table 1:** Baseline characteristics of the six included studies.

**References**	**Country**	**No. of patients** **(No. of positive by predefined criteria, %)**	**Definition of positive CLDN 18.2**	**Immunohistochemical analysis**
(13)	Germany	381 (65, 17%)	IRS > 8	Anti-CLDN 18.2 clone EPR19202 (Abcam, Cambridge, UK, rabbit Mab, dilution: 1:500) and clone 43-14A (Roche Ventana Medical Systems, mouse Mab, dilution: 1:1); FFPE tissue immunostained on a Leica Bond-Max Autostainer (Leica Biosystems, Wetzlar, Germany), with heat-induced epitope retrieval and the Leica Bond HRP Polymer Detection Kit
(14)	Korea	367 (108, 29.4%^a^ or 46, 12.5%^b^)	H-score	Anti-CLDN 18.2 (Abcam, dilution 1:75); FFPE tissue immunostained on a Leica Bond-Max Autostainer, with the Leica Red Refine HRP Polymer Detection Kit
(15)	Germany	481 (203, 42.3%)	H-score	Anti-CLDN 18.2 clone EPR19202 (Abcam, rabbit Mab, dilution: 1:200); FFPE tissue immunostained on a Leica Bond-Max Autostainer, with heat-induced epitope retrieval (ER-2 buffer, Leica, 20 min) and the Leica Refine HRP Polymer Detection Kit
(16)	Korea	82 (12, 14.6%)	Staining was visible in >5% of tumor cells	Anti-CLDN 18.2 rabbit Pab (Thermo Fisher Scientific, Carlsbad, CA, USA, dilution 1:150 with incubation for 15 min at room temperature); FFPE tissue immunostained on a Leica Bond-Max Autostainer, with heat-induced epitope retrieval (pH 6 at 97°C for 20 min) and the Leica Bond Polymer Refine Detection Kit (DS9800)
(17)	Germany	483 (89, 18.4%)	Staining was visible in >5% of tumor cells	Anti-CLDN 18.2 clone EPR19202 (Abcam, cat. no. ab222512, rabbit Mab, dilution: 1:200, incubation for 20 min at 37°C); FFPE tissue immunostained on a Leica Bond-Max Autostainer, with autoclave heat-induced epitope retrieval (Tris-EDTA pH 9 buffer at 121°C for 5 min) and the Leica Bond Polymer Refine Detection Kit for 5 min at 37°C (DS9800)
(18)	Japan	263 (227, 86.6%^c^ or 135, 51.5%^d^)	At least 1+ (weak membrane or cytoplasmic reactivity) intensity in any fraction of tumor cells	Anti-CLDN 18.2 clone 43-14A recognizing the C-terminus of claudin 18 (Ganymed Pharmaceuticals AG, Mainz, DE, mouse Mab, incubation for 30 min at room temperature); FFPE tissue manually immunostained after heat-induced epitope retrieval (10 mM Tris, 1 mM EDTA pH 9 buffer at 95–99°C for 15 min) and a goat anti-mouse horseradish peroxidase conjugated Fab polymer detection system (Nichirei Biosciences, Inc., Tokyo, Japan) for 30 min at room temperature.

*CLDN, claudin; FFPE, formalin fixed paraffin embedded tissue; IRS, immunoreactivity score; H-score, histoscore; Mab, monoclonal antibody; Pab, polyclonal antibody*.

a Positivity was defined as a percentage of staining >10%;

b positivity was defined as a percentage of staining ≥ 51%;

c positivity was defined as at least 1+intensity in any percentage;

d *positivity was defined as a percentage of staining ≥ 40%*.

### Correlation Between CLDN 18.2, Pathological Characteristics, and Prognosis of GC Patients

We conducted the following analysis using the standard meta-analysis to find correlations between CLDN 18.2 and pathological features and prognosis of GC patients. Two subgroups of studies were analyzed according to the definition of CLDN 18.2's positivity and the outcomes were assessed where data were available. The two subgroups were: A (positivity was defined as CLDN 18.2 staining intensity was present in any percentage of tumor cells) and B (positivity was defined as CLDN 18.2 staining intensity was present in more than 40% of tumor cells).

#### By T Clinical Stage

**Figure 2:**
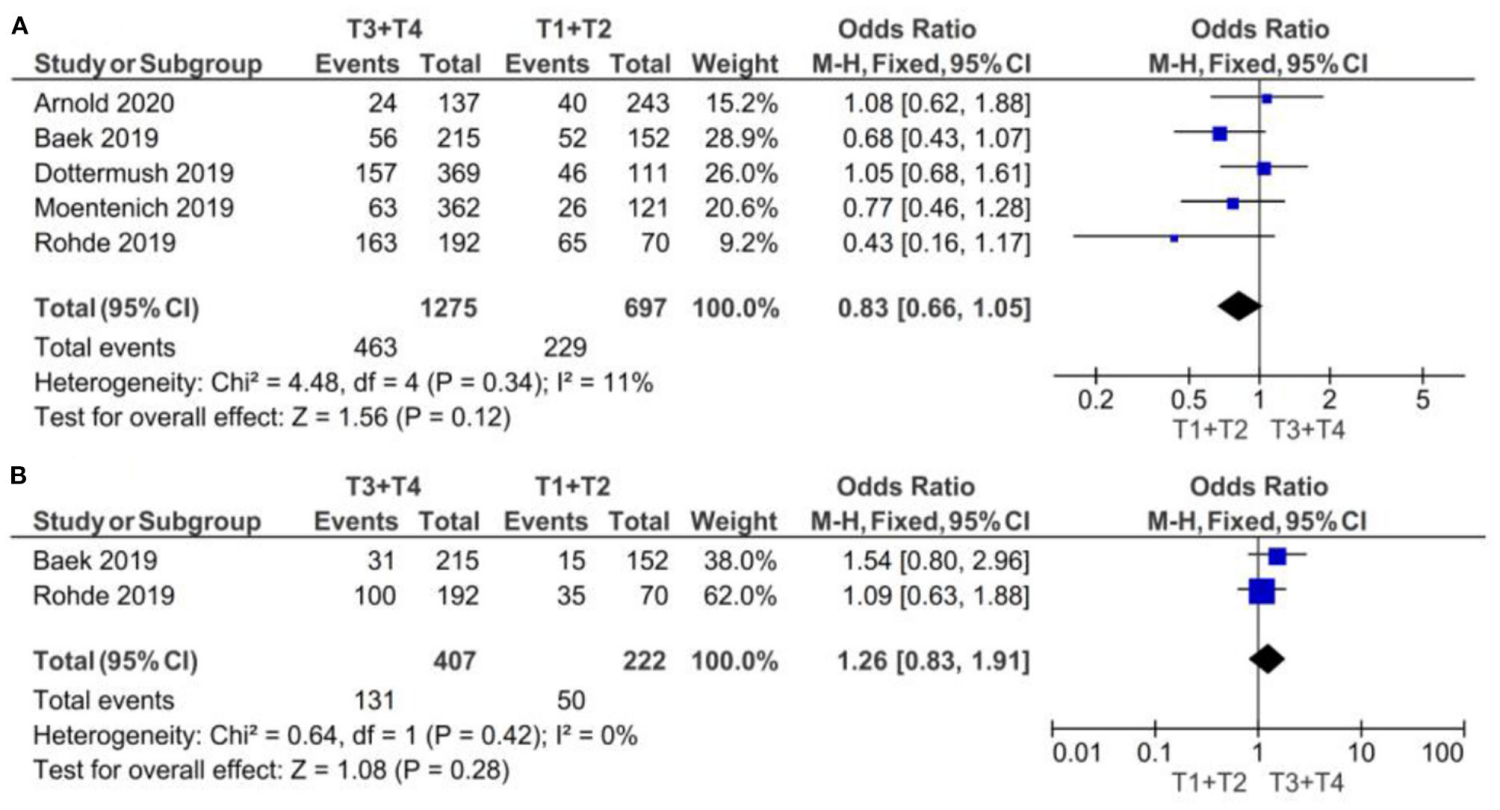
Forest plot on the association between CLDN 18.2 and invasive grade (T3 + T4 vs. T1 + T2). **(A)** The proportion of staining scored in any percentage of tumor cells; **(B)** the proportion of staining scored as ≥40% of tumor cells.

The results are illustrated in the forest plots in Figure 2. If the samples were defined as CLDN 18.2-positive showing specific staining with any fraction of tumor cells, there was no evidence (*p* = 0.12) to indicate correlation between CLDN 18.2 expression and T1 + T2 vs. T3 + T4 clinical stage, with an OR of 0.83 (95% CI 0.66–1.05). The fixed-effect model was used with an I^2^ of 11% (*p* = 0.34) indicating no heterogeneity.

If the samples were defined as CLDN 18.2-positive showing specific staining with more than 40% of tumor cells, there was no evidence (*p* = 0.28) to indicate correlation between CLDN 18.2 expression and T1 + T2 vs. T3 + T4 clinical stage, with an OR of 1.26 (95% CI 0.83–1.91). The fixed-effect model was used with an I^2^ of 0% (*p* = 0.42) indicating no heterogeneity.

The effect estimates and confidence intervals for both individual studies and meta-analysis showed the importance of how CLDN 18.2 was defined as positive. We observed, for example in Baek et al. (14), that the results of OR was 0.68 (95% CI 0.43–1.07) for a positive CLDN 18.2 expression in any percentage staining and 1.54 (95% CI 0.80–2.96) for more than 40% staining. The overall effect was also different in the two subgroups of studies: for subgroup A, but without statistical significance, CLDN 18.2 exhibited more positive expression in patients with T1 + T2 stage than in those with T3 + T4 stage GC; while for subgroup B, but also without statistical significance, CLDN 18.2 exhibited more positive expression in patients with T3 + T4 stage than in those with T1 + T2 stage GC.

#### By N Clinical Stage

**Figure 3:**
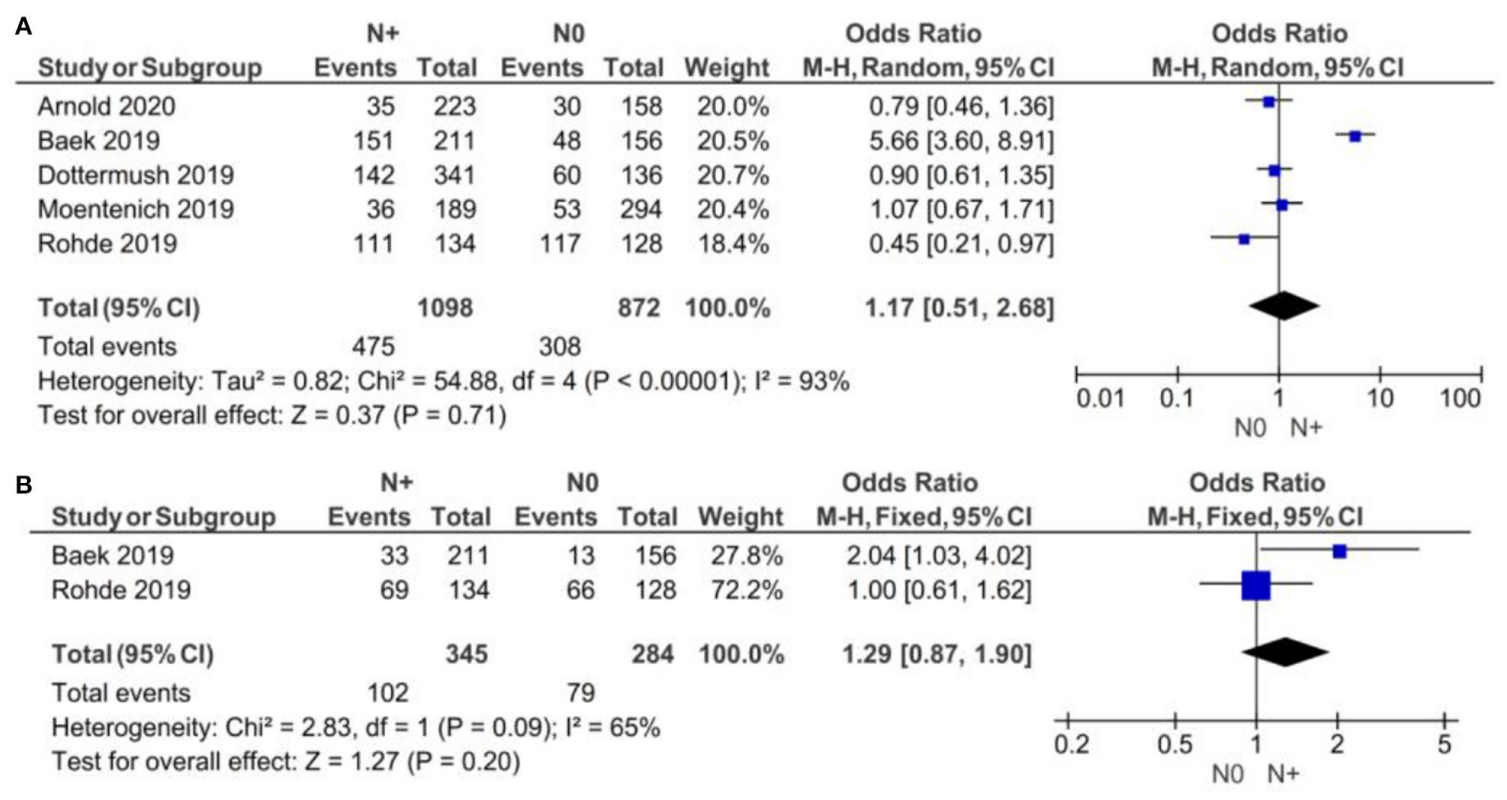
Forest plot on the association between CLDN 18.2 and invasive grade (N+ vs. N0). **(A)** The proportion of staining scored in any percentage of tumor cells; **(B)** the proportion of staining scored as ≥40% of tumor cells.

As demonstrated in Figure 3, no statistically significant correlation was found between positivity of CLDN 18.2 and N clinical stage (N+ vs. N0), neither for subgroup A [*p* = 0.71, with an OR of 1.17 (95% CI 0.51–2.68)] nor for subgroup B [*p* = 0.20, with an OR of 1.29 (95% CI 0.87–1.90)]. We used a random-effect model for the A subgroup with an I^2^ of 93% (*p* < 0.00001) and a fixed-effect model for the B subgroup with an I^2^ of 65% (*p* = 0.09). The high heterogeneity of the A subgroup (Chi^2^ = 54.88) was not observed in subgroup B (Chi^2^ = 2.83).

#### By M Clinical Stage

**Figure 4:**
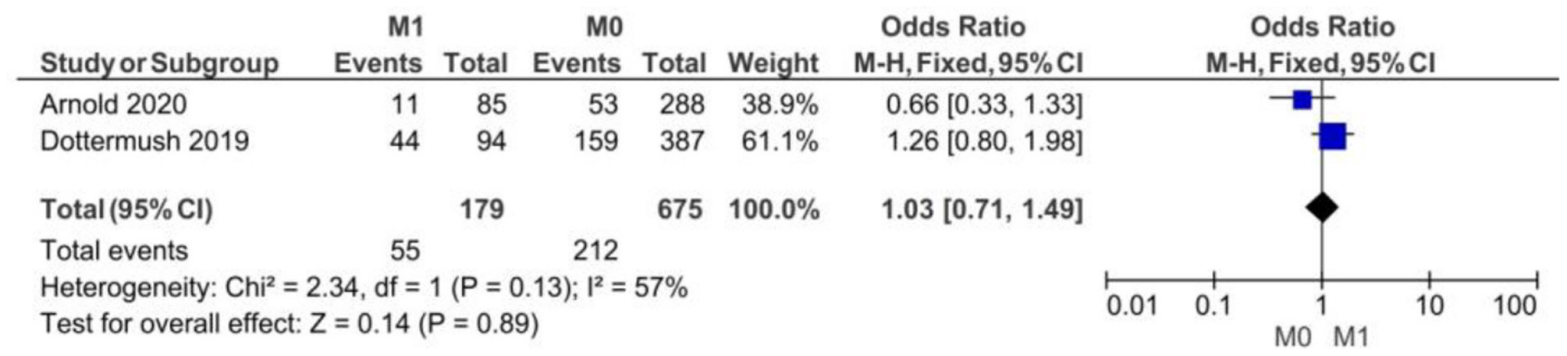
Forest plot on the association between CLDN 18.2 and invasive grade (M1 vs. M0) at the proportion of staining scored in any percentage of tumor cells.

The lack of statistical significance at *p* < 0.05 (*p* = 0.89) proved no correlation between CLDN 18.2 expression and the M clinical stage. The fixed-effect model was used for no heterogeneity of the two studies included in this meta-analysis (*I*^2^ = 57%, *p* = 0.13). The overall effect OR was close to 1 as shown in Figure 4: 1.03 (95% 0.71–1.49).

#### By HER2

**Figure 5:**
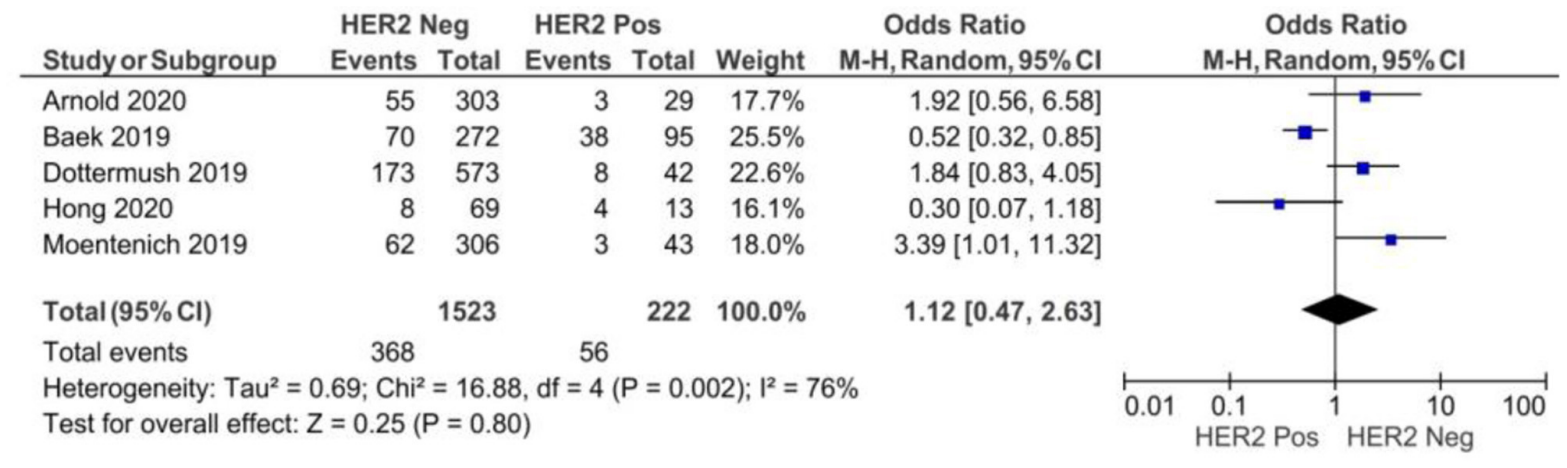
Forest plot on the association between CLDN 18.2 and HER2 at the proportion of staining scored in any percentage of tumor cells.

There were no significant differences between CLDN 18.2 positive and CLDN 18.2 negative GC patients with respect to HER2 statuses, as showed in Figure 5 (*p* = 0.80).

A random-effect model was used for moderate heterogeneity of the five studies included in this meta-analysis (*I*^2^ = 76%, *p* = 0.002). The overall effect OR was 1.12 (95% 0.47–2.63).

#### By Lauren Classification

**Figure 6:**
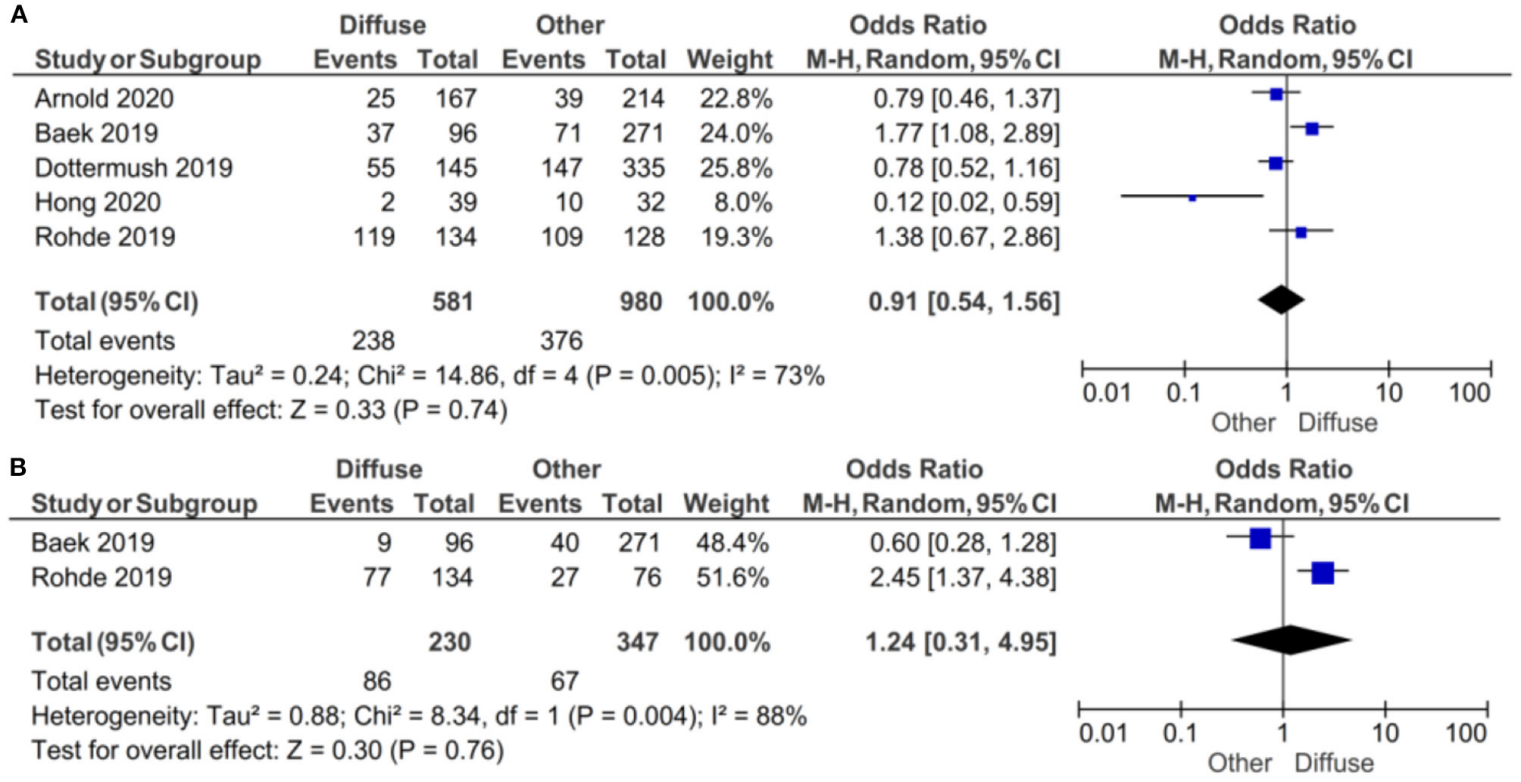
Forest plot on the association between CLDN 18.2 and Lauren classification (diffuse vs. other). **(A)** The proportion of staining scored in any percentage of tumor cells; **(B)** the proportion of staining scored as ≥40% of tumor cells.

If the samples were defined as CLDN 18.2-positive showing specific staining with any fraction of tumor cells (>5 or >10%), there was no evidence (*p* = 0.74) to indicate correlation between CLDN 18.2 expression and diffuse vs. other Lauren classifications, with an OR of 0.91 (95% CI 0.54–1.56), as shown in Figure 6. A random-effect model was used with an I^2^ of 73% (*p* = 0.005) indicating moderate heterogeneity.

If the samples were defined as CLDN 18.2-positive showing specific staining with more than 40% of tumor cells, there was no evidence (*p* = 0.76) to indicate correlation between CLDN 18.2 expression and diffuse vs. other Lauren classifications, with an OR of 1.24 (95% CI 0.31–4.95). A random-effect model was used with an I^2^ of 88% (*p* = 0.004) indicating high heterogeneity.

The effect estimates and confidence intervals for both individual studies and the meta-analysis showed the importance of CLDN 18.2 being defined as positive. We observed, for example in Baek et al. (14), that the result of OR was 1.77 (95% CI 1.08–2.89) for a positive CLDN 18.2 expression in any percentage staining and 0.60 (95% CI 0.28–1.28) for more than 40% staining. The overall effect was also different in the two subgroups of studies.

**Figure 7:**
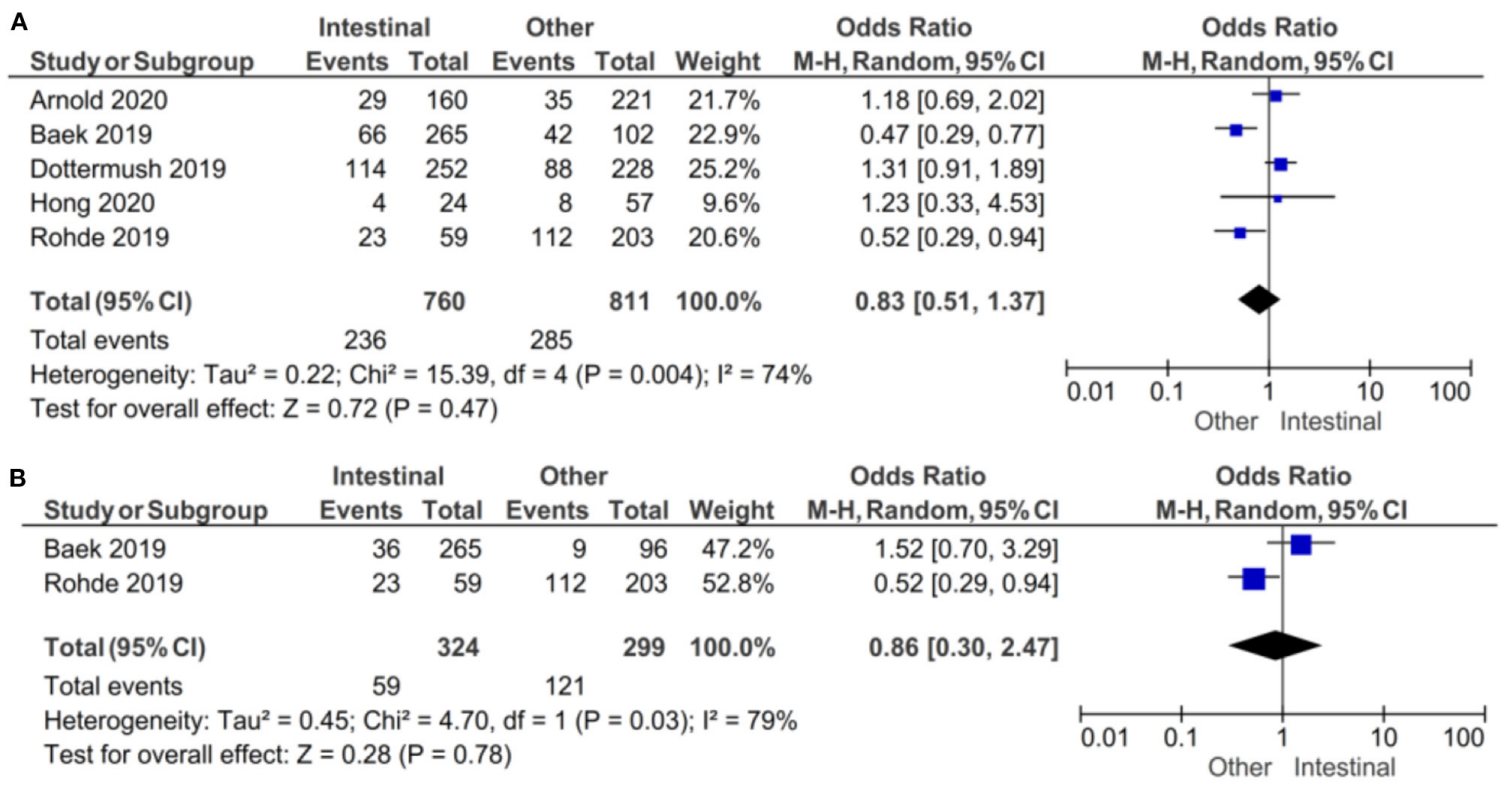
Forest plot on the association between CLDN 18.2 and Lauren classification (intestinal vs. other). **(A)** The proportion of staining scored in any percentage of tumor cells; **(B)** the proportion of staining scored as ≥40% of tumor cells.

In the subgroup of studies where positive CLDN 18.2 was defined as more than 5% staining of tumor cells, there was no evidence (*p* = 0.47) to indicate correlation between CLDN 18.2 expression and intestinal vs. other Lauren classifications, with an OR of 0.83 (95% CI 0.51–2.47). A random-effect model was used with an I^2^ of 74% (*p* = 0.004) indicating moderate heterogeneity, as shown in Figure 7.

In the subgroup of studies where positive CLDN 18.2 was defined as more than 40% staining of tumor cells, there was no evidence (*p* = 0.78) to indicate correlation between CLDN 18.2 expression and intestinal vs. other Lauren classifications, with an OR of 0.86 (95% CI 0.30–2.47). A random-effect model was used with an I^2^ of 79% (*p* = 0.03) indicating moderate heterogeneity.

The effect estimates and confidence intervals for both individual studies and meta-analysis showed the importance of how CLDN 18.2 was defined as positive. We observed, for example in Baek et al. (14), that the results of OR was 0.47 (95% CI 0.29–0.77) for a positive CLDN 18.2 expression of more than 5% staining and 1.52 (95% CI 0.70–3.29) for more than 40% staining. The overall effect was almost the same in the two subgroups of studies.

#### By Grading

**Figure 8:**
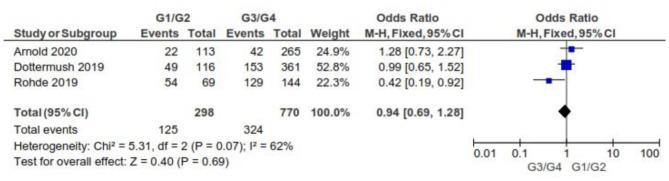
Forest plot on the association between CLDN 18.2 and grading at the proportion of staining scored in any percentage of tumor cells.

There were no significant differences between CLDN 18.2-positive and CLDN 18.2-negative GC patients with respect to grading, as Figure 8 shows (*p* = 0.69).

As for the grading, we found that CLDN 18.2 expression was almost the same in GC tumors with G1/G2 when compared with G3/G4 (OR = 0.94; 95% CI 0.69–1.28). The fixed-effect model was used for no heterogeneity between the three studies included in this meta-analysis (*I*^2^ = 62%, *p* = 0.07).

#### By Overall Survival (OS)

**Figure 9:**
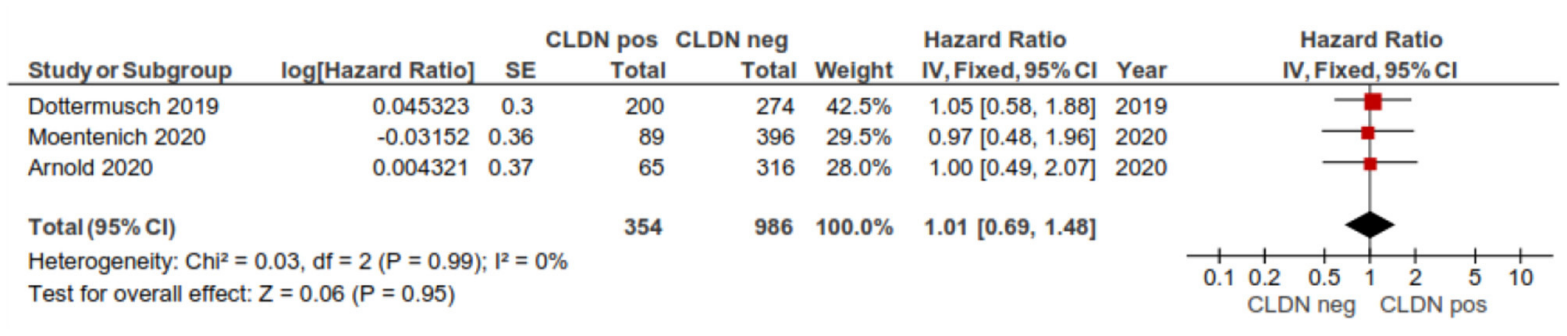
Hazard ratio for OS for patients with positive CLDN vs. negative CLDN.

Three studies were included in the meta-analysis of assessing the hazard ratio (HR) for overall survival (OS) for patients who were CLDN 18.2-positive vs. CLDN 18.2-negative. The fixed-effect model was used (no heterogeneity *I*^2^ = 0% and *p* = 0.99). No significant difference in OS was found between CLDN 18.2-positive and CLDN 18.2-negative: HR = 1.01 (95%CI 0.69–1.48), *p* = 0.95, as Figure 9 shows.

### Publication Bias

**Figure 10:**
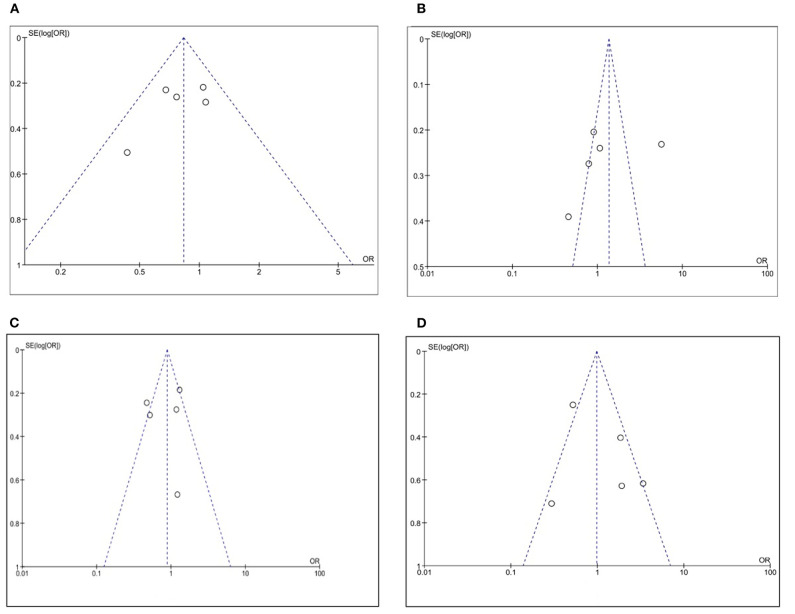
Funnel plot on the association between CLDN 18.2 and pathological characteristics at the proportion of staining scored in any percentage of tumor cells: **(A)** T stage; **(B)** N stage; **(C)** Lauren classification; **(D)** HER2.

Moderately sized and large studies were included in our meta-analysis, as it can be seen in the funnel plots in Figure 10, where no smaller studies appeared toward the bottom of the graph. There was no evidence of any bias because of the observed symmetry: the effect size on the x axis showed that the studies were distributed symmetrically about the mean effect size.

## Discussion

In this meta-analysis, we observed the relationship between CLDN 18.2 expression and GC pathologic features. This tight junction protein CLDN 18.2 is currently considered as a potential target for GC adenocarcinoma and could enlarge the panel of therapeutic options (19). Our results point out that there is no significant connection between CLDN 18.2 and TNM stage, histologic and invasive grade as well as with the Lauren classification.

The claudins are a family of surface proteins which lay the ground for tight cell junctions. Different isoforms are associated with different types of tissue, of which CLDN 18 relates more to GC. CLDN 18.2 is considered a gastric-specific isoform with higher expression in cancer cells than in normal tissue. Generally, it is located within the upper foveolar epithelial cells and is not present within the stem cells areas. However, when carcinogenesis occurs, the tight junctions are disrupted and CLDN 18.2 epitopes are expressed by tumor cells. Thus, this process has proposed the development of a monoclonal antibody against CLDN 18.2 such as zolbetuximab (IMAB362, claudiximab). This new targeted therapy is validated in preclinical studies, and several phase I and II trials are underway with positive results published so far. The FAST study (NCT01630083) (20) showed that combined with first-line chemotherapy, it might improve overall survival (OS) and progression-free survival rate. Zolbetuximab is an IgG1 monoclonal antibody that generates a cascade of processes leading to apoptosis and cell proliferation inhibition. However, it seems to be related so far to higher outcomes only if CLDN 18.2 is expressed in at least 70% of tumor cells (21).

Our meta-analysis reveals that there is no significant correlation between CLDN 18.2 tissue expression and clinicopathologic features. None of the available studies showed any correlation with the TNM stage, however, in T3 + T4 we emphasized a more abundant expression than for T1 + T2, if the positivity of CLDN 18.2 was defined through a higher percentage of stained tumor cells. This was similar for the N stage showing that along with an increased positivity, no correlation was observed (the pooled results showed that CLDN 18.2 was more correlated with the N+ status, in the case of a higher proportion of staining tumor cells).

While our results did not show any positive correlation with Lauren classification, Coati et al. observed that higher prevalence of CLDN 18 had a diffuse type. They also found that higher expression was found in the corpus than the antrum (22).

Regarding the HER2+ status, CLDN 18.2 staining did not correlate with it, even though one study suggested higher expression rates for HER2+ (2+, 3+) statuses (14). On the other hand, two phase III clinical trials (NCT03504397 and NCT03653507) on HER2-negative cases are looking for promising results and might promote CLDN 18.2-directed therapy as a solution for HER2 GC negative patients (23, 24).

Due to the heterogeneity of studies some questions should be raised. First, there is a need for uniformity when differentiating CLDN 18.2 from other variants, currently the only IVD (*in vitro* diagnostics) approved test is the CLAUDETECT 18.2 Kit (developed by Ganymed Pharmaceuticals AG, acquired by Astellas, partnership with Ventana for automated immunohistochemical staining assay on platform). The CLAUDETECT 18.2 Kit was introduced for *in vitro* diagnosis of expression level assessment. This immunohistochemical assay which recognizes the C terminus of claudin 18 is not specific for the isoform 18.2. However, the Anti-CLDN EPR19202 kit (Abcam, Cambridge, UK) is specific for a synthetic peptide within human claudin 18.2 amino acids 1-100, thus it can only detect this isoform. CLDN 18.2 histopathological staining status is important because it will validate patients for future therapy.

However, the cut-off seems to be the key point. Our meta-analysis focused on any percentage of positive staining and > 40% positive cells and showed no correlation with any of the clinicopathologic features, which strongly suggests that standard criteria are yet to be established. Some studies used IRS score or H-score for the definition of positive CLDN 18.2. Perhaps more studies focusing on a higher level of positive staining might obtain better results in relation to TNM stage, grading, as well as OS. This is confirmed by some trial studies which suggest that higher intensity (>75%) will result in better efficacy (longer OS) (20). On the same line of uniformizing the results, it should be mentioned that automated computer-aided image-analysis offers a more objective and reproducible way of quantifying any immunohistochemical staining, for example using parameters like signal area and integrated optical density. Moreover, the advent of multispectral microscopy has opened the avenue for true quantitative staining analysis at the tissue level, a multispectral filter allowing the camera to quantify only the spectral signature of the chromophore has been utilized to visualize the antibody without any interference from the tissue and any counterstaining (25).

Ethnicity represents a main factor in GC response to therapy. The percentage of positive patients varied in both European and Asian countries. While two studies from Germany showed rather similar results with 17 and 18%, Dottermuch et al. (15) had 42% of the patients positive for CLDN 18.2. Results are rather similar in Asia with two Korean studies displaying 15 and 29% positive results and a study from Japan with 87% positive cells. This might emphasize that race involvement in positive staining should be further pursued.

This is the first meta-analysis on CLDN 18.2 and its expression on GC patients. Even though it may represent a new addition for current therapies, our results show a low prevalence with 34.2% in 2,055 patients. The data so far suggest that targeted therapy for CLDN 18.2 could be effective if certain criteria will be established. Clinical trials might help providing more data about the expression of CLD18.2 in assessing claudiximab productivity.

Our results suggest that a new cut-off value for CLDN 18.2 positivity should be taken into account, and that computer generated analysis might be an option for further studies, as it may provide more accurate results. This was actually discussed by clinical trials which achieved better efficacy if higher expression levels were taken into account. Perhaps selecting only patients with high intensity levels and correlated with clinicopathologic data could provide more candidates to establish the therapy candidates.

Our study has some limitations due to the small number of included studies, but it pooled the outcomes for a large number of patients with international findings, recruiting both Caucasians and Asians.

## Conclusion

Even though our results did not show any correlation between CLDN 18.2 staining and the patient's clinicopathologic features, we believe that more specific assays for staining and quantification, as well as a cut-off value for CLDN 18.2 level, might help solve this issue. Hopefully the available trials will shed more light on this new targeted therapy much needed for GC treatment.

## Data Availability Statement

The original contributions presented in the study are included in the article/supplementary material, further inquiries can be directed to the corresponding author.

## Author Contributions

BSU and DIG: conceptualization. BSU and AT-S: methodology. AT-S: software. C-VL, DP, IML, EM, and FB: formal analysis. BSU and VMS: data curation. BSU and AT-S: writing—original draft preparation. DP, DIG, and AS: writing-review and editing. All authors contributed to the article and approved the submitted version.

## Conflict of Interest

The authors declare that the research was conducted in the absence of any commercial or financial relationships that could be construed as a potential conflict of interest.

## Footnotes

^1^GLOBOCAN, https://gco.iarc.fr/today/data/factsheets/populations/900-world-fact-sheets.pdf (accessed November 20, 2020).
